# Greek Sage Exhibits Neuroprotective Activity against Amyloid Beta-Induced Toxicity

**DOI:** 10.1155/2020/2975284

**Published:** 2020-12-07

**Authors:** Antonis Ververis, Georgia Savvidou, Kristia Ioannou, Paschalis Nicolaou, Kyproula Christodoulou, Michael Plioukas

**Affiliations:** ^1^Neurogenetics Department, The Cyprus Institute of Neurology and Genetics, Nicosia 2371, Cyprus; ^2^Cyprus School of Molecular Medicine, The Cyprus Institute of Neurology and Genetics, Nicosia 2371, Cyprus; ^3^Department of Life and Health Sciences, School of Sciences and Engineering, University of Nicosia, Nicosia 2417, Cyprus

## Abstract

Alzheimer's disease (AD) is the most common neurodegenerative disease, affecting the elderly at a high incidence. AD is of unknown etiology and currently, no cure is available. Present medication is restricted to treating symptoms; thus, a need exists for the development of effective remedies. Medicinal plants constitute a large pool, from which active compounds of great pharmaceutical potential can be derived. Various *Salvia* spp. are considered as neuroprotective, and here, the ability of *Salvia fruticosa* (SF) to protect against toxic effects induced in an AD cell model was partly assessed. Two of AD's characteristic hallmarks are the presence of elevated oxidative stress levels and the cytotoxic aggregation of amyloid beta (A*β*) peptides. Thus, we obtained SF extracts in three different solvents of increasing polarity, consecutively, to evaluate (a) their antioxidant capacity with the employment of the free radical scavenging assay (DPPH^•^), of the ferric reducing ability of plasma assay (FRAP), and of the cellular reactive oxygen species assay (DCFDA) and (b) their neuroprotective properties against A*β*_25–35_-induced cell death with the use of an MTT assay. All three SF extracts showed a considerable antioxidant capacity, with the methanol (SFM) extract being the strongest. The results of the total phenolic and flavonoid contents (TPC and TFC) of the extracts and of the FRAP and the DCFDA assays showed a similar pattern. In addition, and most importantly, the dichloromethane (SFD) and the petroleum ether (SFP) extracts had an effect on A*β* toxicity, exhibiting a significant neuroprotective potential. To our knowledge, this is the first report of SF extracts demonstrating neuroprotective potential against A*β* toxicity. In combination with their antioxidant capacity, SF extracts may be beneficial in combating AD and other neurodegenerative diseases.

## 1. Introduction

Alzheimer's disease (AD) is a progressive neurological disorder with a prevalence of 5% among individuals over 65 years old, increasing to 30% among those over 85 years old. It is the commonest form of dementia, as well as the most prevalent neurodegenerative disorder, and is dramatically affecting cognitive and behavioural skills [1]. One of the main contributing factors to AD's progression is the presence of oxidative stress, i.e., a disturbance in the balance between oxidants and antioxidants, in favour of the oxidants. This phenomenon promotes the generation of damaging accumulates of reactive oxygen species (ROS), which contribute to the accumulation of the neurotoxic extracellular amyloid beta (A*β*) plaques in the brain. These deposits further increase oxidative stress, causing more damage to the cells, especially to the neurons that are more vulnerable to the oxidants' activity [2–4].

Presently, there is no available cure for AD, and no significant progress on treatment has been documented in the past 2 decades. Only symptomatic treatment is currently available, mainly with the use of cholinesterase inhibitors, which however are not slowing down AD progression [5]. Consequently, there is a need for novel drugs development that will be more effective in combating AD. A large pool to identify possible candidates from are the medicinal plants and their bioactive compounds [6]. Some of these compounds may have the capacity to counteract the oxidative stress consequences that have been observed not only in AD, but also in every prevalent neurodegenerative disease [7]. So far, a significant number of reports have been published that evaluate the antioxidant potential of various plant extracts and compounds, as well as their ability to ameliorate the A*β* plaques-induced neurotoxicity in AD models, both *in vivo* and *in vitro* [8–13].

The *Salvia* species, commonly referred to as sage, is one of the largest genera belonging to the Lamiaceae family of flowering plants, encompassing around 1000 species [14]. Various studies have shown several *Salvia* species to possess significant antioxidant and anti-inflammatory potential due to their bioactive components such as polyphenols, terpenoids, flavonoids, and other secondary metabolites. Furthermore, *S. officinalis*, *S. miltiorrhiza*, and *S. sahendica* have exhibited neuroprotective properties against A*β*-induced toxicity [9, 11, 15, 16], highlighting a possible use of *Salvia* species in treating AD.

*Salvia fruticosa* Miller (SF), or Greek sage, is an endemic plant of the Eastern Mediterranean Basin of medicinal value in various illnesses of the digestive, of the circulatory, and of other systems [17]. Several studies have also exhibited the antioxidant potential of various Greek sage extracts, which differs depending on the type of plant, the harvesting time, and the extraction methods, with the more polar fractions (ethyl acetate, methanolic, and aquatic–methanolic) showing the strongest antioxidant potential [17–28]. Additionally, Greek sage has been found to possess antifungal, antibacterial, anticancer, anti-inflammatory, and anticholinesterase properties [20, 29–34].

Here, we provide evidence that supports a neuroprotective property for the aerial parts of cultivated SF by alleviating A*β*-induced toxicity. Furthermore, we evaluated the antioxidant potential of the individual fractions derived by the extraction of the aerial parts of the plant, with the use of solvents of increasing polarity.

## 2. Materials and Methods

### 2.1. Chemicals

The solvents methanol, petroleum ether, and dichloromethane; the reagents DPPH^•^, Folin and Ciocalteu's phenol, TPTZ, 20 mM iron(III) chloride solution, sodium acetate, acetic acid, sodium carbonate, sodium nitrite, aluminium chloride, and sodium hydroxide; and the standards used, ascorbic acid, catechin, gallic acid, and Trolox were purchased from Sigma-Aldrich (Taufkirchen, Germany). The standard rutin was purchased from Tokyo Chemical Industry (Tokyo, Japan). DMSO was purchased from Santa Cruz (Heidelberg, Germany).

### 2.2. Plant Material

The Institute of Plant Breeding and Genetic Resources (IPB&GR) of the Hellenic Agricultural Organization “Demeter” provided the SF plant material. The plant populations were cultivated at the experimental field of IPB&GR (40°34'35”N, 21°57'19”E), with the following soil properties: soil type: red loam, pH 7.73, clay: 39.0, organic matter: 1.43%, P_2_O_5_: 45 ppm, and K_2_O: 520 ppm. Mild winters, warm springs, and humid summers characterize the weather at the experimental site. A plant taxonomist conducted the plants identification, while the plants were selected according to their phenotypic and agronomic characteristics, with confirmed genetic material. SF aerial parts were collected prior to blooming, air-dried in the shade before use, and packed in tightly closed containers. The collection process followed the international bioethics guidelines. A voucher specimen was deposited, under the code no. 1215-SlvfELGO, in the Laboratory of Pharmacognosy, Department of Life and Health Sciences, School of Sciences and Engineering, University of Nicosia, for future reference.

### 2.3. Plant Extracts Preparation

Fixed-weight material (56.17 g) of the SF aerial parts was placed into a Soxhlet apparatus 0.6L and was exhaustively extracted with solvents of increasing polarity (petroleum ether, dichloromethane, and methanol), consecutively. The obtained extracts were evaporated under vacuum to dryness. The dry weights of the extracts were 1.03 g (1,83%), 1.13 g (2,01%), and 10.30 g (18,34%), respectively, and were stored in glass tubes until use. For the neuroprotective activity experiments, the extracts were dissolved in DMSO at 250 mg/mL just before use.

### 2.4. DPPH^•^ Radical Scavenging Activity Assay

Radical scavenging activity against the stable radical 1,1-diphenyl,2-picrylhydrazyl (DPPH^•^) (Sigma-Aldrich, Missouri, USA) was conducted as previously described [35], to estimate the antioxidant potential of the three extracts. Serial dilutions of all extracts were prepared. In short, an aliquot of 25 *μ*L of diluted extract was added to 975 *μ*L DPPH^•^ solution (2 × 10^−5^ M) and the mixture was vortexed and kept at room temperature. The decrease in the absorbance was determined at 517 nm, by using a U-2000 Hitachi spectrophotometer (Hitachi, Tokyo, Japan), in a 10 mm quartz cuvette. The absorbance of the DPPH^•^ radical without any extract was measured. The DPPH^•^ concentration in the reaction medium was calculated from the calibration curve. For each extract concentration tested, the percentage of DPPH^•^ remaining in the steady state, was calculated in the following way:

Percentage of remaining DPPH^•^ = [DPPH^•^]at *t* = *T*/[DPPH^•^]at *t* = 0, where *T* is the time necessary to reach the steady state.

The antioxidant capacity of each extract was expressed as the amount of extract necessary to decrease the initial DPPH^•^ concentration by 50% (EC_50_). The antiradical efficiency (AE) is calculated as follows: AE = 1/EC_50_.

### 2.5. FRAP Antioxidant Power Assay

The Ferric Reducing Antioxidant Power was determined as previously described [36]. Appropriately diluted aliquots of the plant extracts (200 *μ*L) were mixed with 1800 *μ*L of freshly prepared FRAP solution. The FRAP solution contained 3.75 mL of 10 mM TPTZ in 40 mM HCl, 3.75 mL of 20 mM iron(III) chloride solution, and 37.5 mL of a 0.3 M acetate buffer (pH 3.6). The mixtures were allowed to stand for 10 minutes at room temperature before the absorbance was measured at 593 nm using a spectrophotometer. The antioxidant activity was expressed as ascorbic acid equivalents (AAE *μ*mol/g sample) and Trolox equivalent antioxidant capacity (TEAC *μ*mol/g sample) using an ascorbic acid and Trolox standard curve.

### 2.6. Determination of Total Phenolics

The total phenolic content (TPC) of the plant extract was determined with the method of Scalbert et al. with slight modifications [37]. 0.5 mL of the extract was mixed with 2.5 mL of a 10-fold diluted Folin–Ciocalteu's phenol reagent and 2 mL of 7.5% sodium carbonate solution in the test tube and shaken vigorously. After 30 min of incubation at room temperature, absorbance was recorded at 760 nm with a spectrophotometer. The total phenolics content in the extract was calculated and expressed as gallic acid equivalents (GAE mg/g sample) using a gallic acid standard curve. Analyses were run in triplicate and the results were expressed as average with the standard deviations.

### 2.7. Determination of Total Flavonoids

The total flavonoid content (TFC) of the plant extract was determined by the aluminium chloride colorimetric method with slight modifications [38]. 0.5 mL of extract or standard solution of catechin or rutin was added to the test tube containing 2 mL of distilled water. 15 *μ*L of 5% sodium nitrite was added to the flask, and after 5 min, 15 *μ*L of 10% aluminium chloride solution was added. After 5 min, 1 mL of 1 M sodium hydroxide was added, and another 1.2 mL of distilled water was added. The solution was mixed well, and the absorbance was measured against the prepared blank at 510 nm using a UV Visible spectrophotometer. Results were calculated with the catechin and the rutin standard curve and recorded as *μ*mol of total flavonoids in a gram of extract, as the catechin (CE) and rutin (RE) equivalents. Analyses were run in triplicate and expressed (total flavonoid content was expressed in *μ*mol of catechin hydrate or rutin/*g* of dry extract) as average values with standard deviations.

### 2.8. Peptides Preparation

A*β*_25–35_ peptides (GenScript, New Jersey, USA) were dissolved in sterile distilled water at 1 mM concentration and were incubated at 37°C for 1 week to allow for aggregates' formation. The aggregated peptides were stored at −20°C until use.

### 2.9. Cell Culture

Human SH-SY5Y cells were routinely cultured in Dulbecco's Modified Eagle's medium supplemented with 10% fetal bovine serum, 5% horse serum, 2 mM Glutamine, 50 U/mL Penicillin, and 50 mg/mL Streptomycin (Thermo Fisher Scientific, Massachusetts, USA) and maintained at 37°C in 5% CO_2_.

### 2.10. DCFDA Assay

To evaluate the antioxidant capacity of the SF extracts in cell culture, the DCFDA/H2DCFDA–Cellular ROS Assay (Abcam, Cambridge, UK) was employed. SH-SY5Y cells were plated at the density of 25,000 cells per well in black, clear-bottomed 96-well plates. The next day, cells were incubated with 20 *μ*M 2',7'-dichlorofluorescin diacetate (DCFDA) for 45 min at 37°C in the dark and then treated with the SF extracts at various concentrations in the presence of 250 *μ*M *tert*-butyl hydroperoxide (TBHP) for 4 hours. DCFDA is a nonfluorescent probe that is oxidized in the highly fluorescent 2',7'-dichlorofluorescein (DCF) in the presence of ROS [39]. DCF fluorescence was measured in a Synergy H1 microplate reader (BioTek, Vermont, USA) at Ex/Em = 485/535 nm. Trolox (500 *μ*Μ) was employed as standard antioxidant.

### 2.11. Cell Viability Determination

To acknowledge any possible neuroprotective effect of the SF extracts, SH-SY5Y cells were plated at the density of 20,000 cells per well in 96-well plates. The next day, cells were incubated with the SF extracts at various concentrations, and after 2h, A*β*_25–35_ were added in a final concentration of 20 *μ*m for 48 hours.

Cell viability was determined using the MTT reduction assay (Abcam). After treatment with extracts, cells were incubated with culture media supplemented with MTT reagent for 3h at 37°C. Then, the culture media supplemented with MTT reagent was removed, and MTT solvent was added to the wells. The plate was covered with foil and was shaken for 15 minutes on an orbital shaker. The absorbance was read at 590 nm in a Synergy H1 microplate reader. Cell viability was calculated as the percentage of the absorbance of the treated cells in relation to the absorbance of control cells. Four independent experiments were conducted.

### 2.12. Statistical Analysis

Results are presented as means ± standard deviation (SD) or means ± standard error of the mean (SEM) of *n* replicates. Statistical significance to compare cell viability differences between control cells and cells treated with the SF extracts, as well as between cells treated with A*β* peptides and cells treated with both A*β* peptides and SF extracts, was determined by one-way analysis of variance (ANOVA) followed by Dunnett's post-hoc test for multiple comparisons. The same analysis determined statistically significant DCF fluorescence differences between cells treated with TBHP and cells treated with TBHP and SF extracts. The level of statistical significance was *p* < 0.05.

## 3. Results

### 3.1. Total Phenolic and Flavonoid Content

The results revealed that the solvents significantly affected the amount of total phenolic and flavonoid contents in the tested extracts. In the present study, the SFM extract had the highest content of phenolics (308,07 mg GAE/g extract) and flavonoids (645,29 *μ*mol CE/g extract and 1069,92 *μ*mol RE/g extract). On the contrary, the SFD extract showed the lowest amount of phenolics (103,84 mg GAE/g extract) and flavonoids (108,15 *μ*mol CE/g extract and 157,45 *μ*mol RE/g extract) (Table 1).

### 3.2. Antioxidant Potential of *Salvia fruticosa* Various Fractions

All the fractions from the plant material were evaluated for their antioxidant activity using various assays. Radical scavenging activity expressed as EC_50_ ranged from 0.336 to 1.477 mg dry extract/mg DPPH^•^. In particular, the SFD fraction possessed the greatest antiradical activity, followed by the SFM. The weakest antioxidant was the SFP fraction. Trolox, known for its good antioxidant activity, was used as standard, and in comparison with the SF extracts, only the SFD extract exceeded approximately half the value of Trolox's antiradical potential (Table 1).

The FRAP assay data are summarized in Table 1. All extracts indicated high capacity to scavenge free radicals varying between 388 and 3093 *μ*mol ascorbic acid/g extract and 419–3217 *μ*mol Trolox/g extract. Contrary to DPPH^•^ assay results, but similar to TCP/TFP, the FRAP assay showed SFM to possess the greatest antiradical activity and SFD to be the weakest antioxidant.

**Figure 1:**
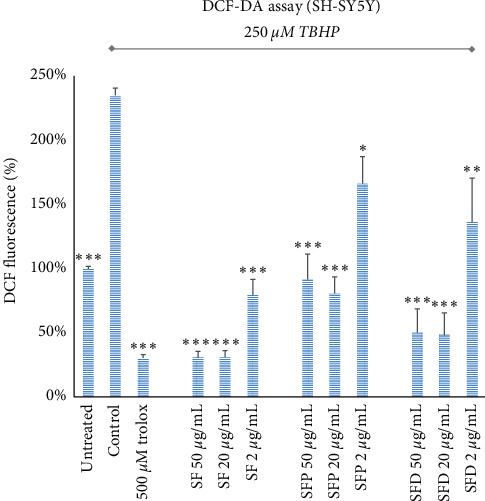
Effect of SF extracts on ROS levels in the presence of TBHP in SH-SY5Y cells. Error bars depict the SEM of four independent experiments. ^*∗*^ refers to *p* < 0.05, ^*∗∗*^ to *p* < 0.01, and ^*∗∗∗*^ to *p* < 0.001 statistical significance, compared with control cells that were treated with 250 *μ*m TBHP.

Similar results to the FRAP assay were produced with the DCFDA assay where the ability of the various SF extracts to scavenge ROS in living cells was demonstrated (Figure 1). All three SF extracts of this study were able to significantly reduce ROS presence in SH-SY5Y cells treated with TBHP, with the most efficient one being the SFM. The optimal SF concentrations for each extract, in terms of antioxidant activity, were 50 and 20 *μ*g/mL. In lower extracts' concentrations (2 *μ*g/mL), ROS levels appear higher, showing a dose-dependent antioxidant impact of the extracts. Remarkably, in the presence of the oxidant TBHP, treatment with SFM (at 50 or 20 *μ*g/mL), or SFD (at 50 or 20 *μ*g/mL), dropped ROS levels much lower than of cells not treated with TBHP.

### 3.3. Neuroprotective Activity of *Salvia fruticosa* Various Fractions

**Figure 2:**
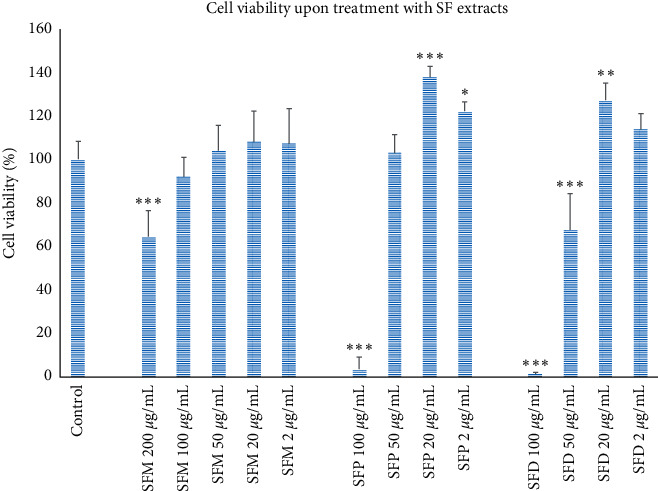
Effect of treatment with various SF extracts concentrations on SH-SY5Y cell viability. Error bars depict the SD of four independent experiments. ^*∗*^ refers to *p* < 0.05, ^*∗∗*^ to *p* < 0.01, and ^*∗∗∗*^ to *p* < 0.001 statistical significance, compared with untreated cells.

For the better evaluation of the possible application of SF extracts in AD treatment, their neuroprotective activity was assessed. This took place in SH-SY5Y cells, and initially, the cytotoxic effect of the extracts on these cells was evaluated (Figure 2). The results showed that treatment with SF extracts in high concentrations (SFM extract ≥ 200 *μ*g/mL, SFP extract ≥ 100 *μ*g/mL, and SFD extract ≥ 50 *μ*g/mL) reduced cell viability. Conversely, at the concentrations of 20 and 2 *μ*g/mL, the SFP extract had a statistically significant effect on improving cell viability (1.38- and 1.22-fold, resp., in comparison to control cells). A 1.27-fold increase in cell viability was also observed upon treating with 20 *μ*g/mL SFD. At 2 *μ*g/mL SFD treatment, there was an obvious trend for cell viability increase, which however was not statistically significant.

**Figure 3:**
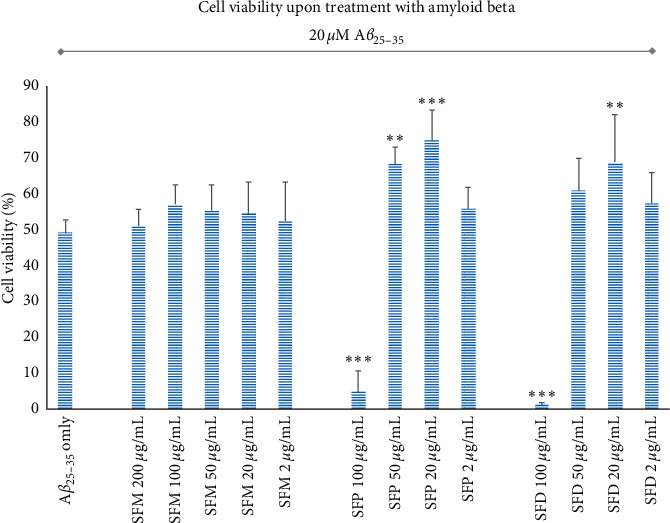
Cytoprotective effect of various SF extracts concentrations on A*β*-induced toxicity in SH-SY5Y cells. Error bars depict the SD of four independent experiments. ^*∗∗*^ refers to *p* < 0.01, and ^*∗∗∗*^ refers to *p* < 0.001 statistical significance, compared with A*β*_25-35_ only treated cells.

Upon treatment with 20 *μ*m A*β*_25–35_ for 48h, SH-SY5Y cells displayed ∼50% viability compared to control. Pretreatment of SH-SY5Y cells with the SFP and the SFD extracts managed to significantly reduce the A*β*_25–35_ toxic effects and partly rescued cell viability (Figure 3). The most prominent effect was observed with the SFP extract, which at 20 *μ*g/mL restored cell viability at ∼75% of control cells and at 50 *μ*g/mL restored cell viability at ∼69% of control cells. At 2 *μ*g/mL, the SFP extract showed a trend to rescue cell viability, while at 100 *μ*g/mL the toxic effects of SFP dropped cell viability to almost zero.

A similar pattern was observed for the SFD extract (Figure 3). Pretreatment with 20 *μ*g/mL partly rescued cell viability to ∼69% with statistical significance. A trend for rescue was recorded at 50 and 2 *μ*g/mL of SFD. It should be noted that while treatment with 50 *μ*g/mL SFD extract reduced cell viability in normal cells, it increased cell viability in cells treated with the neurotoxic A*β*_25–35_, revealing a struggle between the neuroprotective capability and the cytotoxic effect of SFD extract. In the smaller, nontoxic concentration of 20 *μ*g/mL, the SFD extract exhibits the maximum neuroprotective potential and shows no signs of cytotoxicity.

Finally, the SFM extract did not display any statistically significant rescue of A*β*_25–35_-treated cells at the tested concentrations range, even though a small increase was recorded at every condition (Figure 3). Further increase of the SFM concentration above 200 *μ*g/mL is not expected to help, because of the cytotoxic effect the SFM exhibits in high concentrations (Figure 2).

## 4. Discussion

To our knowledge, the present study has investigated for the first time the neuroprotective potential of various *Salvia fruticosa* extracts, in relation to A*β*-induced neurotoxicity. The *in vitro* AD model employed in this study was SH-SY5Y human bone marrow neuroblastoma cells treated with A*β*_25–35_ peptides. A*β*_25–35_ is the shortest fragment generated *in vivo* from the proteolysis of A*β*, which at the same time maintains the toxicity of the larger peptides [40, 41]. In conjunction with SH-SY5Y cells, A*β*_25–35_ peptides have been used extensively to assess the neuroprotective effects of various plant extracts and plant-derived substances [11, 42–44]. The solvents used for the SF extracts generation were of increasing polarity: petroleum ether (nonpolar), dichloromethane (moderately polar), and methanol (polar). Since the chemical complexity of plant extracts, polarity, different functional groups, and chemical behaviour may lead to scattered results due to the antioxidant test employed [45], the evaluation of the antioxidant activity of the extracts was examined with more than one method: the DPPH^•^ assay [46] and the FRAP antioxidant power assay [36]. The FRAP assay appears to be simple, attractive, and potentially useful test, due to inexpensive reagents, highly reproductive results, and fast procedures. On the other hand, the DPPH^•^ radical scavenging activity assay is capable of the evaluation of the antioxidant potential of both hydrophilic and lipophilic compounds and can afford data on the reduction potential of the sample, even in cases where the structure of the electron donor is not known (e.g., plant extracts) [47]. In addition, the DCFDA assay was employed to assess the antioxidant capacity of the extracts in living cells [39].

Environmental conditions, genetic and other factors may affect the yield, the chemical composition, and the biological activities of the plant extracts [48, 49]. Therefore, this study used cultivated plants with superior phenotypic and agronomic characteristics, instead of native plants that show large variations in morphological features and phytochemical components, thus affecting polyphenolic composition. Moreover, taking into account that the quality of plant material depends on its phytochemical content, which is largely affected by intrinsic and external parameters, cultivation provides the standardization of the starting plant material of constant high quality, ensuring high yields of the desired constituents [49, 50].

The values obtained from the assays for the determination of the total phenolic and the total flavonoid content revealed that these contents varied in range between the SF extracts, due to the usage of the different solvents used for the extraction of the chemical compounds. The SFM extract had the highest content of phenolics and flavonoids, while the SFD had the lowest. Overall, the values obtained for the determination of the TPC and the TFC revealed a high yield of phenolics and flavonoids, due to the possible presence of rosmarinic acid, carnosol, apigenin, and luteolin, that have been previously identified as the most abundant constituents of SF extracts from the experimental field that our material is originating from [23].

The FRAP assay results showed that SFM possesses the greatest antiradical activity and SFD is the weakest antioxidant; thus, FRAP followed a trend similar to that of TPC and TFC. Additionally, the DCFDA assay conducted in cells, corresponded with the FRAP/TPC/TFC results, in showing the SFM extract to possess the highest ROS scavenging ability. The DCFDA assay demonstrated that SFD and SFP exhibit a considerable antioxidant capacity as well. The superiority of SFM extract in terms of antioxidant capacity, as well as the similar trend of TPC/TFC and FRAP assay results, is in agreement with previously published findings [23, 29]. On the other hand, TPC and TFC did not show a similar pattern with the DPPH^•^ results, since DPPH^•^ assay showed the SFD extract to possess the strongest antioxidant activity, followed by the SFM and then by the SFP extract. This discordance of TPC/TFC and DPPH^•^ in *S. fruticosa* has been also reported in a previous work, where the highest DPPH^•^ scavenging ability of the chloroform extract corresponded to the lowest TPC, in comparison to the other extracts of that study [20]. These observations demonstrate that, in addition to polyphenols, other constituents may also contribute to the antioxidant activities of medicinal plants and act as radical scavengers.

These findings confirm the difficulty in evaluating the antioxidant potential of a plant tissue or product by using only one single assay and highlight the need to employ various methods to truly uncover the antioxidant capacity of a sample, possibly due to the different mechanisms underlying each assay [51]. In addition, based on our information, this is the first time that the SF extracts antioxidant capacity is evaluated in terms of ROS reduction in living cells. Nevertheless, the approaches used here (DPPH^•^, FRAP, and DCFDA) confirm that the SF extracts of this study exhibit important antioxidant activity and add to the previous reports of SF extracts of various regions possessing similar properties [17, 18, 20–27, 29]. This antioxidant potential of SF has been attributed, thus far, to the presence of several phenolic acids and flavonoids at high levels, of known antioxidant properties, such as rosmarinic acid, carnosol, apigenin, and luteolin [20, 21, 23, 52–58].

The neuroprotective effects experiments showed that the SFP extract exhibits the highest neuroprotection capacity against amyloid beta toxicity. A smaller but similar effect was shared by the SFD extract. Unfortunately, the SFM extract did not reverse the A*β*_25–35_ consequences. These results may lead to the assumption that SF extracts obtained with the use of less polar solvents possess higher neuroprotective ability, but further investigation with a larger number of solvents is necessary before safe conclusions may be drawn.

According to this study, specific SF extracts display a statistically important neuroprotective effect in concentrations of 20 to 50 *μ*g/mL (the SFD at 20 and 50 *μ*g/mL and the SFP at 20 *μ*g/mL). In smaller concentrations, the protective effect against A*β* neurotoxicity tends to fade away, while in higher concentrations cytotoxic effects antagonize with the extracts' neuroprotective potential. Similar competition phenomena in relatively high concentrations have been observed in other plant species extracts, such as extracts derived from *Frankenia thymifolia*, *Caliphruria subedentata*, *Piper sarmentosum*, and *Sasa senanensis* [10, 59–61].

As documented here, the SF extracts exhibit a cytotoxic effect at relatively high concentrations. This effect of SF was previously described to be more intense in various human cancer cell lines than noncancer cells; thus, SF extracts hold promise for cancer treatment [24, 31, 62]. At lower SF extracts concentrations, we observed neuroprotective potential, a pro-proliferative effect, and antioxidant activity. These properties make SF extracts candidates for inclusion in medicinal remedies for the treatment of AD and other neurodegenerative diseases.

*S. fruticosa* is the fourth *Salvia* species found to have protective properties against A*β* neurotoxicity [9, 11, 15, 16], and this potential has been attributed to *Salvia* adequacy in active constituents such as flavonoids, terpenoids, and phenolic acids [63]. In essential oils and extracts obtained from SF grown in various areas of Greece, Italy, Jordan, and Lebanon, many substances of these categories were identified [20–22, 62–66]. Methanolic extracts obtained from SF grown at the experimental field of IPB&GR, from which our study material was collected, were rich in phenolic acids such as rosmarinic acid and benzoic acid derivatives, in the terpenoids 1,8-cineole, carnosic acid, and carnosol, and in the flavonoids apigenin and luteolin, all of which are known neuroprotective substances [9, 23, 67–71]. The above active compounds are most likely present in the SF extracts of this study, and their presence in the three different extracts (SFD, SFM, and SFP) in different proportions can explain the differences in neuroprotective capacity between the extracts. However, the fact that SFD and SFP exhibit important neuroprotectivity, but have a lower TPC and TFC comparatively to the nonneuroprotective SFM, hints that the substances responsible for the neuroprotectivity in SF are not restricted to the polyphenols or the flavonoids categories, and those additional active substances may be present. Further phytochemical analysis, with the isolation and the structure elucidation of such compounds in every extract, may clarify the above points.

Oxidative stress is involved in the progression of the commonest neurodegenerative diseases, such as AD, Parkinson's disease, amyotrophic lateral sclerosis, and others [72]. In AD, increased oxidative stress promotes the generation of senile plaques formed by A*β* peptides, and these plaques contribute to further elevation of oxidative stress [2–4]. Thus, a holistic approach to treat AD needs to focus on both antioxidant and neuroprotective aspects, and medicinal remedies that combine these properties may be considered more advantageous. Based on this assumption, between the three extracts of the current study, the most promising for AD treatment are the SFD and the SFP extracts that combine an important antioxidant potential and a significant neuroprotective activity against A*β* toxicity. Nevertheless, SFM can also be of medicinal use for its antioxidant capacity, while there is always the possibility of developing a mixture of several extracts to capitalize on all the benefits that different SF extracts have.

## 5. Conclusions

Conclusively, the present study showed for the first time that SF extracts exhibit a pronounced neuroprotective effect against A*β* toxicity. In combination with their significant antioxidant potential shown here and by others as well [17, 18, 20–27, 29], and their known anticholinesterase ability [20, 33, 34], SF extracts can prove useful for the treatment of AD and of other neurodegenerative diseases.

## Figures and Tables

**Table 1:** Total phenolic content, total flavonoid content, and antioxidant potential of the three extracts from SF aerial parts, as calculated with the employment of DPPH^•^ and FRAP assays. Results are presented as average ± SD.

		SFM	SFP	SFD
TPC	mg *GAE/*g extract	308.07 ± 38.85	164.37 ± 32.01	103.84 ± 14.13

TFC	*μ*mol *CE/*g extract	645.29 ± 56.62	550.12 ± 162.16	108.15 ± 21.91
	*μ*mol *RE/*g extract	1069.92 ± 82.03	898.17 ± 260.55	157.45 ± 28.19

DPPH^•^	*EC*_*50*_^*A*^	0.51 ± 0.01	1.48 ± 0.03	0.34 ± 0.01
	*AE* ^*B*^	1.96	0.68	2.97

FRAP	*μ*mol *AAE/*g extract	3093.18 ± 451.12	579.62 ± 95.39	388.17 ± 81.11
	*μ*mol *TEAC/*g extract	3217.67 ± 484.90	605.03 ± 103.11	419.38 ± 94.87

^**A**^Efficient concentration (mg antioxidant/mg DPPH^•^): amount of antioxidant needed to decrease the initial DPPH^•^. concentration by 50%. ^B^Antiradical efficiency: 1/EC_50_. AETrolox (DPPH^•^): 5.59.

## Data Availability

The datasets used and/or analyzed during the current study are available from the corresponding author upon reasonable request.
